# Marine Plastics from Norwegian West Coast Carry Potentially Virulent Fish Pathogens and Opportunistic Human Pathogens Harboring New Variants of Antibiotic Resistance Genes

**DOI:** 10.3390/microorganisms8081200

**Published:** 2020-08-07

**Authors:** Vera Radisic, Priyank S. Nimje, André Marcel Bienfait, Nachiket P. Marathe

**Affiliations:** 1 Institute of Marine Research, 5005 Bergen, Norway; veraradisic96@gmail.com (V.R.); priyankn21@gmail.com (P.S.N.); andre.marcel.bienfait@hi.no (A.M.B.); 2 Department of Biological Sciences, University of Bergen, 5006 Bergen, Norway

**Keywords:** marine plastics, microplastics, fish pathogens, antibiotic resistance, Norway, *Aeromonas* sp.

## Abstract

To our best knowledge this is the first study characterizing fish pathogens isolated from marine plastics from the West coast of Norway for their potential for pathogenicity using whole genome sequencing. Marine plastic polymers identified as polyethylene, polyethylene/ethylene vinyl acetate copolymer and polypropylene, yielded a total of 37 bacterial isolates dominated by *Pseudomonas* spp. (70%). Six isolates representing either fish pathogens or opportunistic human pathogens were selected for whole genome sequencing (WGS). These included four isolates belonging to *Aeromonas* spp., one *Acinetobacter beijerinckii* isolate and one *Morganella morganii* isolate. Three *Aeromonas salmonicida* isolates were potentially virulent and carried virulence factors involved in attachment, type II and type VI secretion systems as well as toxins such as *aerA/act, ahh1, ast, hlyA, rtxA* and *toxA. A. salmonicida* and *Acinetobacter beijerinckii* carried new variants of antibiotic resistance genes (ARGs) such as β-lactamases and chloramphenicol acetyltransferase (*catB*), whereas *Morganella morganii* carried several clinically relevant ARGs. Our study shows that marine plastics carry not only potentially virulent fish pathogens but also multidrug resistant opportunistic human pathogens like *M. morganii* and may serve as vectors for transport of these pathogens in the marine environment.

## 1. Introduction

Plastics are synthetic, anthropogenic materials made from a wide range of organic polymers such as polyethylene (PE), polypropylene (PP), polystyrene (PS), polyvinyl chloride (PVC), polyurethane (PUR), polyethylene terephthalate (PET), polybutylene terephthalate (PBT) and polyamide (PA), commonly known as nylon [1]. Plastic materials enter the marine ecosystem in many different ways, such as being dumped, lost [2], carelessly handled [2,3] or left [2,4]. Plastics found in the environment are mostly classified into three different categories based on their sizes: nanoplastic (<100 nm), microplastic (100 nm–5 mm) and macroplastic (>5 mm) [5,6,7].

Pollution with plastics and accumulation of plastics in the oceans has a big impact on the marine ecosystem [8,9,10]. Ingestion of plastic by marine organisms can disturb their energy balance, affect behavior and sometimes block the intestinal tract leading to sublethal effect or, in the worst-case, cause death [11,12]. Small microplastic (<10 µm) and nanoplastic particles appear to have an increased plastic particle toxicity to aquatic life [13]. Plastic can also be toxic due to the breakdown products as well as due to the presence of chemicals absorbed or released from plastics, such as plasticizers [14,15,16]. Although some polymers are biodegradable, albeit at a very slow rate, others are not [1] and thus plastics stay in the environment for a long time.

Plastic provides a hydrophobic surface for the attachment of microbes and hence different microbes colonize plastics in the marine environment [5,17,18]. Therefore, marine plastics may function as vectors for the transport of bacteria, including fish pathogens, in the oceans [19]. Colonization of plastics by bacteria can also facilitate horizontal gene transfer of antibiotic resistance genes (ARGs), owing to biofilm formation [20]. Accordingly, a study has shown presence of multidrug resistant bacteria on plastics from marine environments [5].

Plastic pollution has been reported in the marine environment in Norway [21]. To date, Norway is a country with a low burden of antibiotic resistance in clinics and in the environment [22]. Although this is true, there is lack of knowledge about microbiota associated with plastic found in the marine environment in Norway as well as associated ARGs. The aim of our study was to investigate the presence of fish pathogens and antibiotic resistant bacteria on marine plastics collected from the West coast of Norway. We isolated fish pathogens and opportunistic human pathogens from plastic surfaces, analyzed whole genome sequences of these isolates and report their potential for pathogenicity as well as new variants of ARGs.

## 2. Materials and Methods

### 2.1. Sample Collection

Macroplastic samples (*n* = 7), with different sizes ranging from one centimeter to 15 cm, were collected from the intertidal zone at beaches in Øygarden, Vestland county, Norway. The coordinates for the sampling areas are 60°29′57.6″ N 4°55′05.3″ E and 60°29′54.5″ N 4°54′52.6″ E, respectively. The samples were collected in either sterile 50 mL tubes or sterile plastic bags. These samples were then stored at 4 °C and transported back to the lab for analysis.

### 2.2. Isolation and Identification of Bacterial Strains

Plastic samples were carefully washed with sterile phosphate buffer saline (PBS) before making suspensions. Suspensions were made by adding 10 mL of sterile PBS to each plastic sample in 50 mL sterile tubes. Four sterile glass beads (4 mm diameter) were added to the tubes, followed by vortexing for 90 s at maximum speed. Serial dilutions of the suspension were prepared in sterile PBS and spread on Mueller–Hinton agar plates and MacConkey agar plates, both media containing either ampicillin (100 µg/mL), cefotaxime (10 µg/mL) or no antibiotics. Mueller–Hinton agar plates were incubated at 25 °C for 24–48 h, while MacConkey agar plates were incubated at 37 °C for 24–48 h. A total of 37 colonies were picked and purified by restreaking on Mueller–Hinton agar with ampicillin. Fish pathogens like *Aeromonas* spp. and *Vibrio* spp. are intrinsically resistant to penicillins, thus, ampicillin facilitated selection of fish pathogens [23,24]. All isolates were stored as glycerol stocks at −80 °C for further use. For identification, isolates were grown on Mueller–Hinton agar with ampicillin at 30 °C for 24 h. The strains were typed using a Bruker MALDI Biotyper at Veterinær instituttet (Bergen, Norway) using the MALDI Biotyper database.

### 2.3. Genomic DNA Extraction and Illumina Sequencing

Genomic DNA was extracted from isolates cultivated overnight on Mueller–Hinton agar with ampicillin using QlAamp Fast DNA Stool Mini Kit (Qiagen, Hilden, Germany) following the manufacturer’s instructions. NanoDrop 1000 and Qubit 2.0 (Thermo Scientific, Waltham, MA, USA) were used to quantify the extracted DNA. Extracted DNA was sent for sequencing to the Norwegian Sequencing Centre (Oslo University Hospital, Ullevål, Oslo, Norway). Nextera DNA Flex Library Prep Kit (Illumina, San Diego, CA, USA) was used for preparing sequencing libraries. Sequencing was performed on an Illumina MiSeq platform (Illumina, San Diego, CA, USA), using 2 × 300 bp chemistry.

### 2.4. Genome Sequence Assembly and Screening for ARGs

Adapters were removed from the obtained raw reads and the reads were quality filtered using BBDuk (version 38.75; https://jgi.doe.gov/data-and-tools/bbtools/bb-tools-user-guide/). Sequences were assembled in SPAdes (version 3.13.0) [25] using the default parameters; spades.py −1 xx_R_1__trim.fastq −2 xx_R_2__trim.fastq --careful --only-assembler --cov-cutoff auto -o output_name. Genome annotation was performed using National Center for Biotechnology Information (NCBI) Prokaryotic Genomes Automatic Annotation Pipeline (PGAAP) [26]. The genome sequences were screened for antibiotic resistance genes using the CARD database (version 3.0.7) [27] and ResFinder 3.2 database [28]. The virulence genes were identified using VFanalyzer and the virulence factor database (VFDB) [29]. Genome sequences have been submitted to GenBank under the following genome accession numbers: JAACGC000000000, JAACGB000000000, JAACGA000000000, JAACFZ000000000, JAACFY000000000, JAACFX000000000 and JAACFW000000000, respectively.

### 2.5. Phylogenetic Analysis

Amino acid sequences for CphA, RtxA and AerA were extracted from the genome sequences of *Aeromonas* spp. Additional sequences for respective proteins were downloaded from GenBank (www.ncbi.nlm.nih.gov/genbank). The sequences were aligned using clustalX version 2.1 [30]. The phylogenetic tree was generated by the neighbor joining method using MEGA-X with bootstrap analysis (1000 replicates) [31].

### 2.6. Antibiotic Susceptibility Testing

Isolates were grown on Mueller–Hinton agar with ampicillin at 30 °C overnight and were used for making suspensions for determination of minimum inhibitory concentration (MIC). MICs were determined for cefotaxime (CT), tetracycline (TC), ciprofloxacin (CI), ampicillin (AM), meropenem (MP), streptomycin (SM), trimethoprim (TR), gentamicin (GM), imipenem (IP) and chloramphenicol (CL) using E-test according to manufacturer’s protocol (bioMérieux, Paris, France). *Escherichia coli* strain CCUG 17,620 was used as a control strain for E-test quality check.

### 2.7. Identifying Plastic Polymer Type

The polymer type for plastic samples was identified by attenuated total reflection infrared spectroscopy (Cary670 FTIR spectrometer, Agilent Technologies) equipped with a monolithic diamond crystal unit (GladiATR™, PIKE Technologies, Madison, WI, USA). Spectra were collected over the wavenumber range 440–4000 cm^−1^. The samples were analyzed by 32 co-added scans, with a resolution of 8 cm^−1^. The spectra were compared to spectra of known standards using a library of environmental relevant synthetic and natural polymers [32], and commercial libraries for polymers (Bio-Rad Sadtler) in the KnowitAll Informatics System software (BioRad Laboratories, Hercules, CA, USA). Each polymer type was determined by a combination of the best-fitted spectra and the expertise of the operator on interpreting polymer IR spectra.

## 3. Results

### 3.1. Identification of the Plastic Polymers and Bacterial Strains

**Figure 1:**
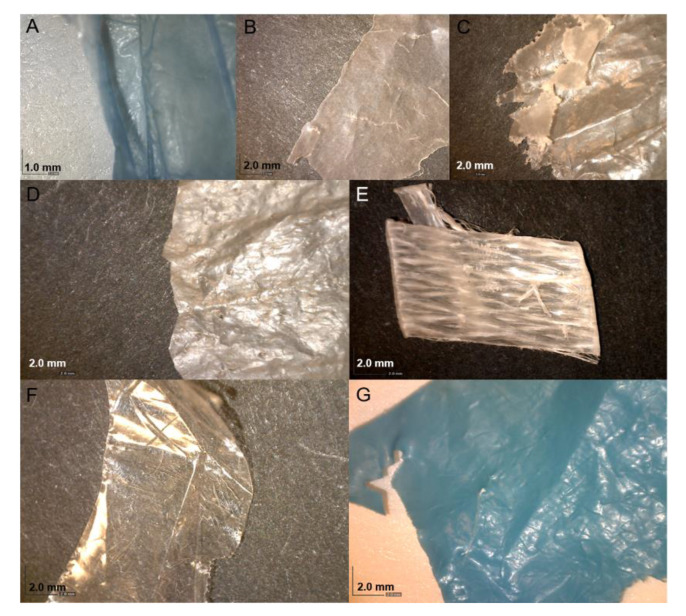
Images of samples collected in this study. (**A**,**C**,**D**,**F**,**G**) are polyethylene; (**B**) is polyethylene/ethylene vinyl acetate copolymer; (**E**,**F**) are polypropylene.

The plastic polymers were identified as polyethylene, polyethylene/ethylene vinyl acetate copolymer or polypropylene (Figure 1). A total of 37 isolates were obtained from the samples (Table S1). Only two isolates were obtained from polypropylene, while the rest were found on polyethylene or polyethylene/ethylene vinyl acetate copolymer. *Pseudomonas* spp. dominated the isolates (26 of 37). Four isolates representing fish pathogens *Aeromonas* spp. and two isolates representing opportunistic human pathogens (*Morganella morganii* and *Acinetobacter beijerinckii*, respectively) were chosen for characterization with whole genome sequencing (WGS). Genome sequence assembly statistics and GenBank accession number for the genome sequences of the isolates are represented in Table S2. Sequencing coverage ranged from 55× to 110×.

### 3.2. Antibiotic Resistance and ARGs

All three *A. salmonicida* isolates were resistant to ampicillin. *M. morganii* and *Ac. beijerinckii* were multidrug resistant, with resistance against at least three different classes of antibiotics (Table 1). Class C β-lactamases and chloramphenicol acetyltransferase (*catB*) were detected in all six isolates. Class B2 metallo-β-lactamase *cphA* was detected in three *A. salmonicida* isolates, with a new variant of *cphA* (≤98% identity) present in two of the three *A. salmonicida* isolates (Table 2 and Figure S1). Along with *cphA* variants all *Aeromonas* isolates carried *qnrA* gene. Isolate 2HC4 (*Ac. beijerinckii*) carried new variants of four different ARGs including a class A and a class C β-lactamase, aminoglycoside acetyltransferase and chloramphenicol acetyltransferase. *M. morganii* carried several resistance genes with 100% homology to previously described ARGs. The list of ARGs detected in the genome sequences of these isolates is presented in Table 2.

### 3.3. Virulence Factors

**Figure 2:**
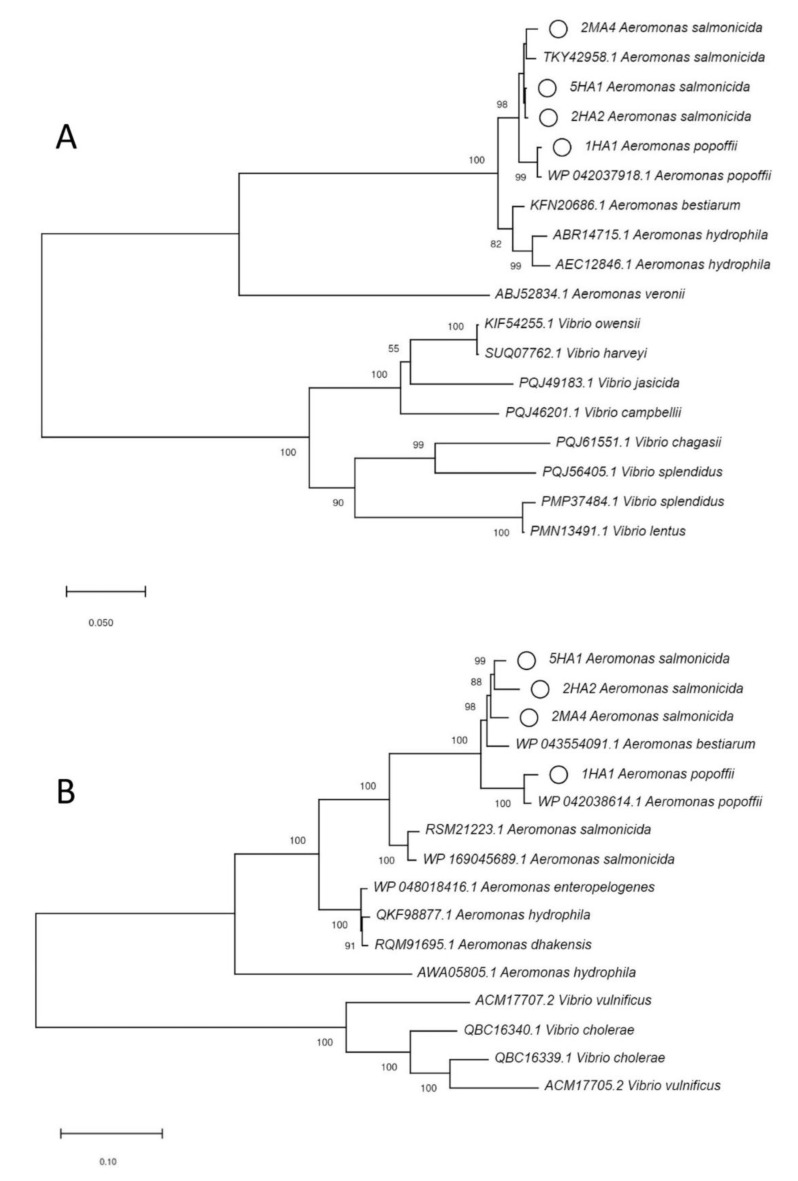
(**A**) Phylogenetic tree of AerA based on amino acid sequences. (**B**) Phylogenetic tree of RtxA based on amino acid sequences. The proteins highlighted with small circles are found in *A. salmonicida* isolates in this study. Accession numbers and host organisms are presented for sequences downloaded from GenBank. Numbers at nodes are bootstrap values (%) based on 1000 resampled datasets; only values >50% are given.

All three *A. salmonicida* isolates had genes for virulence factors involved in attachment, type II (T2SS) and type VI (T6SS) secretion systems as well as toxins such as *aerA/act, ahh1, ast, hlyA, rtxA, rtxB, rtxC, rtxD, rtxE, rtxH* and *toxA.* This suggests that they have potential for causing infection. *M. morganii* carried genes involved in acid resistance, genes involved in iron uptake, and type six secretion system proteins. A phylogenetic tree of RtxA and AerA proteins from *A. salmonicida* isolates and closely related proteins from other species is presented in Figure 2. A list of virulence genes detected in all sequenced isolates is presented Table S3.

## 4. Discussion

To our best knowledge, this is the first study analyzing genome sequences of multidrug resistant bacteria, including fish pathogens, associated with plastic found in the marine environment in Norway. Using 16S rDNA clone libraries, Viršek et al. [33] suggested that microplastics might serve as a vector for the transport of pathogenic bacteria, especially fish pathogen *A. salmonicida*. Our study strengthens the notion that fish pathogens as well as other opportunistic human pathogens are present on marine plastics. We further show the presence of virulence genes and new variants of antibiotic resistance genes in *A. salmonicida* isolated from these plastics.

Most *Aeromonas* spp. are opportunistic pathogens and are widespread in the marine environments [34,35]. *Aeromonas* spp. are known to cause infection in both humans and animals [36]. *A. salmonicida* is one of the most important fish pathogens [37], causing disease in healthy wild and cultured stocks of salmon and other fish species [36]. All *A. salmonicida* isolates in this study carried virulence factors like the *rtxA* toxin. Repeats in toxins (RTX) *rtxA* is a cytotoxin that is an important virulence factor for many fish pathogens, including *Vibrio* spp. and *Aeromonas* spp. It disrupts host cells membranes, aiding pathogenesis [38,39]. Along with *rtxA*, other cytotoxins like aerolysin *aerA* [40], extracellular hemolysin *ahh1*, heat-stable cytotonic enterotoxin *ast*, hemolysin III and *hylA* were detected in these isolates [41]. A study suggests that the presence of both *aerA* and *ahh1* represents the most cytotoxic genotype in *A. hydrophila* [41]. In addition, *Aeromonas* isolates also carried Exotoxin-A (*toxA*), which is a major virulence factor in *Pseudomonas aeruginosa* [42]. Two *A. salmonicida* isolates (2HA2 and 2MA4) carried genes involved in immune evasion (capsular polysaccharide), phagocytosis prevention (*rmlC, wbfU, wbfY*) and endotoxin from *Bordetella*. In addition to these, several others virulence factors like adhesion factors, T2SS [43,44] and T6SS [45,46] were detected. The presence of virulence factors in the genome does not necessarily guarantee expression of these virulence factors and further in-depth studies are needed to establish the virulence of these isolates. Nevertheless, the presence of these virulence genes indicates that these isolates have potential for causing infections. This emphasizes the risk posed by plastics as vectors for the transport of potentially virulent fish pathogens in the marine environment, especially in Norway where aquaculture is one of the major activities.

All three isolates of *A. salmonicida* in our study carried resistance genes like *ampC, bla*_OXA_*, cphA, qnrA* and *catB.* New variants of *cphA* (Class B2 metallo-β-lactamase) were present in the two isolates (5HA1 and 2MA4) of *A. salmonicida* whereas new variants of FOX/MOX family class C β-lactamase (96.3% to 98.4% identity) were detected in all three isolates. Marine bacteria have been shown to be a source of ARGs found in the clinics [47,48,49]. A recent study by Ebmeyer et al. [50] showed that *Aeromonas* spp. are the origin of several clinically important β-lactamases like CMY-1/MOX-family AmpC β-lactamases MOX-1, MOX-2 and MOX-9. Similarly, another study from Ebmeyer et al. [51] showed that FOX AmpC β-lactamases originated in *A. allosaccharophila*. They proposed that the mobilization and fixation of these genes may be recent and may have happened during the antibiotic era. Plastics provide a hydrophobic surface for the attachment of microbes promoting colonization and biofilm formation [52]. In accordance, an increased frequency of resistance plasmid transfer in bacteria associated with microplastics was observed [20]. Plastics also absorb a range of pollutants including antibiotic and heavy metals that are known to be drivers of antibiotic resistance [5,53,54,55]. This may create selection pressure, aiding transfer and/or mobilization of ARGs in the surface associated microbiota on marine plastics [56]. In order to better understand the role of marine plastics in mobilization and selection of ARGs, more research is thus warranted.

We detected opportunistic human pathogens like *M. morganii* [57,58,59], *Ac. beijerinckii* [60] and *A. popoffii* [61] on plastic surfaces. *M. morganii* previously belonged to family *Enterobacteriaceae,* which also consists of classical human pathogens like *Escherichia coli*, *Salmonella typhi* and *Klebsiella pneumoniae*. It was recently reclassified to be included in its own family *Morganellaceae* [62]. Although reclassified, *M. morganii* is an important emerging opportunistic human pathogen causing a variety of infections ranging from wound infections, urinary tract infections to meningitis [57,58,59]. *M. morganii* isolate (2MA1) carried virulence genes involved in acid resistance (*ureB*, *ureG*), genes involved in iron uptake, and T6SS proteins. Further, this isolate was resistant to tetracycline, chloramphenicol and β-lactams as well as carried several clinically important resistance genes like *tetD*, *aac3* and *catB* [59]. The presence of multi-drug resistant human associated bacteria carrying multiple ARGs suggests that plastic may serve as vectors for transport of not only fish pathogens but also opportunistic human pathogens in the marine environment. Our results are in accordance with a recent study that showed presence of potentially pathogenic bacteria on plastic debris from Guanabara Bay in Brazil [63].

Aquaculture is both historically and economically important for Norway. Norway is considered the world’s second largest exporter of seafood after China and delivers fish to more than 100 countries [64,65]. In 2018, more than 1.35 million ton of fish, mostly salmon (*Salmo salar*) (1.28 million ton) and rainbow trout (*Oncorhynchus mykiss*) (68,345 ton) were farmed in Norway, with a first-hand value of 67.8 billion NOK (8.34 billion USD) [66,67]. The presence and spread of potentially virulent fish pathogens on marine plastics, could have a major impact on aquaculture in Norway. Hence, more research on understanding of the role of plastic surface-associated microbial communities and their biogeochemical functions in the marine environment is needed, especially in Norway.

## 5. Conclusions

Our study demonstrates the presence of fish pathogens and human associated bacteria on marine plastics from Norway. We show the potential for pathogenicity of *A. salmonicida* isolates obtained from marine plastics, with presence of genes encoding toxins, hemolysins and adhesion factors in their genome sequences, as well as describe new variants of ARGs carried by plastic associated bacteria. Our study strengthens the notion that plastic debris may serve as vectors for transport for fish pathogen as well as other opportunistic human pathogens in the marine environment. Marine plastics colonized by potentially virulent fish pathogens may impact aquaculture. Hence, in-depth follow-up studies for better understanding the role of plastic in the spread of antibiotic resistant pathogens in the marine environment are needed.

## Figures and Tables

**Table 1:** Minimum inhibitory concentration (MIC) of different antibiotics.

Isolate	Name	MIC (µg/mL)										Isolation Source
		CT	TC	CI	AM	MP	SM	TR	GM	IP	CL	
2MA1	*Morganella morganii*	0.023	256	0.008	>256	0.032	4	0.125	0.75	0.50	24	Polyethylene/Ethylene vinyl acetate copolymer
5HA1	*Aeromonas salmonicida*	0.023	0.25	0.003	>256	0.032	4	0.032	0.75	0.25	0.38	Polyethylene
2HA2	*Aeromonas salmonicida*	0.047	0.50	0.002	>256	0.023	8	0.25	1.5	0.25	1.0	Polyethylene/Ethylene vinyl acetate copolymer
2MA4	*Aeromonas salmonicida*	0.094	0.38	0.003	>256	0.032	8	0.50	1.5	0.38	0.75	Polyethylene/Ethylene vinyl acetate copolymer
1HA1	*Aeromonas popoffii*	0.064	0.19	<0.002	>256	0.004	6	0.125	0.75	0.047	0.38	Polyethylene
2HC4	*Acinetobacter beijerinckii*	12	1.5	0.094	>256	0.50	2	6	1.0	0.19	24	Polyethylene/Ethylene vinyl acetate copolymer

Legend: CT (cefotaxime), TC (tetracycline), CI (ciprofloxacin), AM (ampicillin), MP (meropenem), SM (streptomycin), TR (trimethoprim), GM (gentamicin), IP (imipenem) and CL (chloramphenicol).

**Table 2:** Overview of the different antibiotic resistance genes (ARGs) detected in whole genome sequence of the isolates.

Isolate	Name	Gene	Closest Blast Hit	Percent Identity (Amino Acid)
2MA1	*Morganella morganii*	*bla*_DHA_	DHA family class C β-lactamase	100.00%
		*tet(D)*	tetracycline efflux MFS transporter Tet(D)	100.00%
		*aac(3)*	aminoglycoside 3-N-acetyltransferase	100.00%
		*catB*	antibiotic acetyltransferase	100.00%
5HA1	*Aeromonas salmonicida*	*ampC*	FOX/MOX family class C β-lactamase	98.43%
		*bla*_OXA_	class D β-lactamase	100.00%
		*cphA*	CphA family subclass B2 metallo-β-lactamase	97.64%
		*qnrA*	Qnr family pentapeptide repeat protein	100.00%
		*catB*	antibiotic acetyltransferase	98.64%
2HA2	*Aeromonas salmonicida*	*ampC*	FOX/MOX family class C β-lactamase	97.90%
		*cphA*	CphA family subclass B2 metallo- β-lactamase	99.61%
		*bla*_OXA_	class D β-lactamase	100.00%
		*qnrA*	Qnr family pentapeptide repeat protein	100.00%
		*catB*	antibiotic acetyltransferase	98.64%
2MA4	*Aeromonas salmonicida*	*ampC*	FOX/MOX family class C β-lactamase	96.33%
		*bla*_OXA_	class D β-lactamase	99.24%
		*cphA*	CphA family subclass B2 metallo-β-lactamase	96.85%
		*qnrA*	Qnr family pentapeptide repeat protein	100.00%
		*catB*	antibiotic acetyltransferase	99.09%
1HA1	*Aeromonas popoffii*	*bla*_OXA_	class D β-lactamase	99.62%
		*ampC*	FOX/MOX family class C β-lactamase	98.95%
		*qnrA*	Qnr family pentapeptide repeat protein	100.00%
		*catB*	antibiotic acetyltransferase	99.10%
2HC4	*Acinetobacter beijerinckii*	*ampC*	class C β-lactamase	96.46%
		*bla*	class A β-lactamase	96.11%
		*aac(6′)-I*	AAC(6′)-Ighjkrstuvwx family aminoglycoside N-acetyltransferase	97.93%
		*catB*	antibiotic acetyltransferase	96.24%

## Data Availability

Genome sequences have been submitted to GenBank under the following genome accession numbers: JAACGC000000000, JAACGB000000000, JAACGA000000000, JAACFZ000000000, JAACFY000000000, JAACFX000000000 and JAACFW000000000.

## Supplementary Materials

The following are available online at https://www.mdpi.com/2076-2607/8/8/1200/s1, Figure S1: Phylogenetic tree for *cphA* β-lactamases based on the amino acid sequence. The proteins highlighted with circles are new variants found in *A. salmonicida* isolates in this study. Accession numbers are presented for sequences downloaded from GenBank. PFM1 (QDC33502.1) representing subclass B2 metallo-beta-lactamase; PFM-1 from *Pseudomonas fluorescens* is used as an out group for construction of phylogenetic tree. Numbers at nodes are bootstrap values (%) based on 1000 resampled datasets; only values >50% are given. Table S1: Identification and isolation source of the isolates obtained in the study. Table S2: Genome sequence assembly statistics for the whole genome sequences of the six isolates. Table S3: A list of virulence genes detected in the genome sequence of the isolates.
